# Editorial: Advances in Heart Transplantation

**DOI:** 10.3389/fimmu.2022.960800

**Published:** 2022-06-27

**Authors:** Guido Moll, Zhenhua Dai, Niels Olsen Saraiva Câmara

**Affiliations:** ^1^ Berlin Institute of Health (BIH) Center for Regenerative Therapies (BCRT) and Berlin-Brandenburg School for Regenerative Therapies (BSRT), Berlin Institute of Health (BIH) at the Charité Universitätsmedizin Berlin, corporate Member of Freie Universität Berlin, Humboldt-Universität zu Berlin, Berlin, Germany; ^2^ State Key Laboratory of Dampness Syndrome of Chinese Medicine, The Second Clinical College of Guangzhou University of Chinese Medicine, Guangzhou, China; ^3^ Section of Immunology and Joint Immunology Program, Guangdong Provincial Academy of Chinese Medical Sciences, Guangzhou, China; ^4^ Guangdong Provincial Key Laboratory of Clinical Research on Traditional Chinese Medicine Syndrome, Guangzhou, China; ^5^ Guangdong-Hong Kong-Macau Joint Lab on Chinese Medicine and Immune Diseases, Guangzhou University of Chinese Medicine, Guangzhou, China; ^6^ Department of Immunology, Institute of Biomedical Science, University of São Paulo, São Paulo, Brazil; ^7^ Nephrology Division, Department of Medicine, Federal University of São Paulo, São Paulo, Brazil

**Keywords:** heart transplantation, heart failure, transplant rejection, immune tolerance, biomarkers/new targets

## Introduction

**Figure 1:**
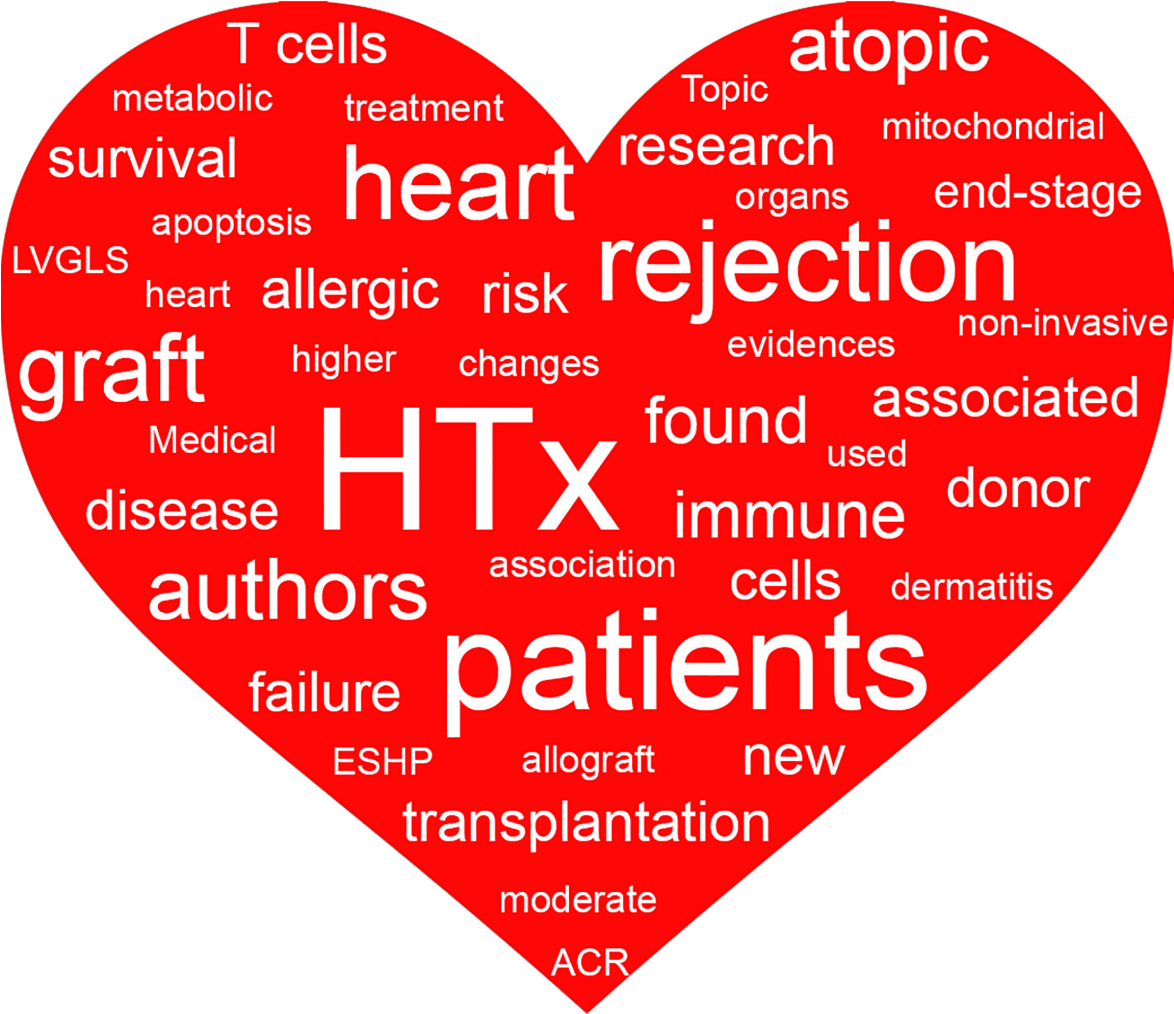
Word cloud of the most frequent terms. This picture describes the most commonly used words in the Editorial on the Research Topic Advances in Heart Transplantation. Note that the size of the words is proportional to their frequency.

Heart transplantation (HTx) is considered a standard therapy for patients with end-stage heart failure refractory to medical treatment. Currently, the one-year and 10-year survival is around 85% and 60%, respectively, thus calling for further improvements in short- and long-term outcomes (1). The number of patients with end-stage heart failure is increasing, while the limited number of available donor organs is still a major limiting factor to the broader applicability of HTx (2). Advancements in HTx to improve quality of life and survival of higher risk patients, including those in waiting list, with new methods, such as broadening the donor pool and making the best use of existing organs by supportive means are anticipated. A better understanding of organ graft immune acceptance and connection between heart and other organs will be decisive for any attempt to impr5ove graft survival (3). The subjects covered within this Research Topic include contributions on progress made in the field of HTx and related fields, such has heart failure diagnosis, risk factors, treatment, prognosis, graft immune response and novel treatment approaches in HTx. These include innovations in heart failure and HTx, potential new drugs to be used in HTx, new targets to control transplant rejection or enhance immune tolerance (or graft acceptance), strategies for expanding the donor pool for heart transplantation, new important clinical evidence advances in HTx, and association between previous disease and graft rejection after HTx. Here, we summarize the 7 manuscripts that were submitted to this Research Topic (**Figure 1**).

## Understanding and Modulating Heart Allograft Rejection

Kakuda et al. from the Department of Cardiovascular Medicine at the University of Tokyo in Japan present a case report of acute cellular rejection (ACR) associated with atopic dermatitis exacerbation 3 years after HTx in a 32-year-old man with progressing atopic dermatitis admitted to regular monitoring of graft rejection. Little evidence exists on the potential association between atopic/allergic disease and graft rejection in solid organ transplantation (SOT), but this case suggested an association between atopic disease and graft rejection. Consequently, the authors examined 76 patients who underwent HTx at their hospital and found that patients with atopic/allergic disorders, such as atopic dermatitis and asthma, tended to have a higher frequency of moderate rejection than non-allergic patients (P=0.012). The authors suggest that exacerbation of allergic disease might cause graft rejection of the transplanted organ, and that it is important to carefully evaluate the risk of graft rejection if there is a previous history of atopic/allergic disease.

In two mechanistic studies, Chinese groups studied the mechanisms underlying heart allograft rejection and new therapeutic approaches to modulate the outcome. Xu et al. from Tongji Medical College found that ablation of Survivin (encoded by *Birc5* gene) in T cells attenuates allograft rejection after murine heterotopic HTx by inducing apoptosis in T cells and thus shifting the ratio of Th1 and Treg cells. In turn, Ma et al. from the Fujian Provincial Key Laboratory of Organ and Tissue Regeneration at the School of Medicine and Organ Transplantation Institute of Xiamen University in China found that administration of moderate doses of Berberine (5 mg/kg) – a traditional Chinese medicine known to induce tumor cell apoptosis – prolongs mouse heart allograft survival by activating T cell apoptosis *via* the mitochondrial pathway without any obvious Berberine-related toxicity. Berberine was found to eliminate alloreactive T cells and to reduce CD4+ and CD8+ T cell infiltration.

## Cardiac Biomarkers in Heart Transplantation

Clemmensen et al. from the Department of Cardiology at Aaarhus University Hospital in Denmark evaluated the relevance of left ventricular global longitudinal strain (LVGLS) and non-invasive cardiac biomarkers for acute cellular rejection (ACR) diagnosis and severity assessment in HTx patients. All HTx patients transplanted between 2013 to 2019, including 1436 endomyocardial biopsies (EMBs) from 83 HTx patients, were included. The authors found that patients with ≥2R ACR have lower LVGLS and higher Troponin T and N-terminal pro brain natriuretic peptide (Nt-ProBNP) than patients without 2R rejection. A non-invasive model combining changes in LVGLS and Troponin T or Nt-ProBNP showed excellent negative predictive value and moderate sensitivity and may be used as a gatekeeper to invasive biopsies after HTx.

Kamath et al. from the Division of Cardiology at Los Angeles Medical Center (UCLA) in the United States evaluated the variability in donor-derived cell-free DNA scores (dd-cf-DNA) as non-invasive liquid (blood) biomarker to predict mortality in HTx recipients in a single-center retrospective pilot study, including 72 adult HTx patients with at least 3 concurrent AlloMap/AlloSure results. A total of 5 patients (6.9%) died during the median follow-up of 480 days. Given the small size of the patient cohort, the authors carefully suggest that increased variability of dd-cf-DNA in HTx patients is associated with both mortality risk and the presence of donor specific antibodies (DSA).

## Heart Allograft Suitability for Transplantation and Heart Valve Immune Privilege

Olkowicz et al. from the University of Toronto and Toronto General Hospital in Canada found that dynamic metabolic changes during prolonged *ex situ* heart perfusion (ESHP) are associated with myocardial decline. The authors used minimally invasive solid-phase micro-extraction (SPME) microprobes sampling and global metabolite profiling to evaluate changes in metabolic patterns associated with myocardial functional decline during ESHP in porcine and human hearts. The authors observed striking metabolic alterations during prolonged 8-hours ESHP associated with uncontrolled inflammation, endothelial injury, increased mitochondrial oxidative stress, disruption of mitochondrial bioenergetics, and accumulation of harmful lipid species. This method may be of use to differentiate between donor hearts transplantable from those that should be discarded.

Hill et al. from the Divisions of Cardiothoracic and Transplant Surgery at the Medical University of South Carolina in the United States reflect on the “Immune Privilege of Heart Valves”. The authors state that immune privilege is an evolutionary adaptation that protects vital tissues with limited regenerative capacity from collateral damage by the endogenous immune response, with some classical examples including the anterior chamber of the eye and the brain, but also the placenta, testes, and articular cartilage. Here, the authors review the experimental and clinical evidence from heart valve transplantation that indicates that heart valves are spared from alloimmune injury and may thus be considered as immune privileged sites.

## Conclusion

This multidisciplinary Research Topic has brought together specialists in Immunology, Transplantation and Biomarker Research, to share their knowledge on current scientific and therapeutic strategies to advance HTx. The different articles contained in this collection reflect the great interest, knowledge, and innovation in these fields.

## Author Contributions

All authors listed have made a substantial, direct, and intellectual contribution to the work and approved it for publication.

## Funding

Contributions from GM were made possible by the German Research Foundation (DFG) and the German Federal Ministry of Education and Research (BMBF) through the BSRT (GSC203) and BCRT, respectively, and in part supported by the European Union’s Horizon 2020 research and innovation program (Horizon 2020 Framework Program) under the grant agreements no. 733006 (PACE) and no. 779293 (HIPGEN). ZD was supported in part by grants from the State Key Laboratory of Dampness Syndrome of Chinese Medicine (SZ2021ZZ16, SZ2021ZZ43, and SZ2021ZZ18), Guangdong Provincial Key Laboratory of Clinical Research on Traditional Chinese Medicine Syndrome (ZH2020KF02), National Natural Science Foundation of China (81603717, 81873261), Guangzhou University of Chinese Medicine (Grant No: AFD018171Z11099), and Special Fund of Guangdong Provincial Science and Technology Innovation Strategy (Guangdong-Hong Kong-Macau Joint Lab, 2020B1212030006). NC was supported in part by grants from the Fundação de Amparo à Pesquisa do Estado de São Paulo (FAPESP, São Paulo, Brazil) (grant numbers 2014/50833-1 and 2017/05264-7), Conselho Nacional de Desenvolvimento Científico e Tecnológico (CNPq) and Coordenação de Aperfeiçoamento de Pessoal de Nível Superior (CAPES, financial code 001). This work was also supported under the International Collaboration Research Funding from FAPESP and The Netherlands Organization for Scientific Research (NWO, The Netherlands, Grant number 2019/19435-3).

## Conflict of Interest

The authors declare that the research was conducted in the absence of any commercial or financial relationships that could be construed as a potential conflict of interest.

## Publisher’s Note

All claims expressed in this article are solely those of the authors and do not necessarily represent those of their affiliated organizations, or those of the publisher, the editors and the reviewers. Any product that may be evaluated in this article, or claim that may be made by its manufacturer, is not guaranteed or endorsed by the publisher.

## References

[1] Masha L. Geographic Variation in US Heart Transplant Outcomes and Consequences for Health Care Equity. JAMA Netw Open (2020) 3:e2028856. doi: 10.1001/jamanetworkopen.2020.28856 33295968

[2] Bakhtiyar SS Godfrey EL Ahmed S Lamba H Morgan J Loor G. Survival on the Heart Transplant Waiting List. JAMA Cardiol (2020) 5:1227–35. doi: 10.1001/jamacardio.2020.2795 PMC7675100 32785619

[3] Millington TM Madsen JC. Innate Immunity and Cardiac Allograft Rejection. Kidney Int Suppl (2010) S18-21. doi: 10.1038/ki.2010.417 PMC3261228 21116311

